# Excessive Visit-to-Visit Small and Dense Low-Density Lipoproteins Elevate Cerebral Small Vessel Disease Progression Risk in the Elderly

**DOI:** 10.3389/fneur.2022.851735

**Published:** 2022-06-29

**Authors:** Weike Liu, Jing Xu, Huajing Song, Chunju Zhang, Yanli Yao, Hua Zhang, Yue-Chun Li, Zhendong Liu

**Affiliations:** ^1^Department of Cardiology, Second Affiliated Hospital and Yuying Children's Hospital of Wenzhou Medical University, Wenzhou, China; ^2^Department of Cardiology, Shandong Provincial Hospital Affiliated to Shandong First Medical University, Jinan, China; ^3^School of Basic Medicine, Shandong First Medical University and Shandong Academy of Medical Sciences, Jinan, China

**Keywords:** lipid, variability, Apolipoprotein E, risk factor, cerebral small vessel disease

## Abstract

**Objective:**

Small and dense low-density lipoprotein (sdLDL) elevation may be among the most sensitive early biomarkers for nascent cardiovascular disease. This study, therefore, investigated the association between visit-to-visit changes in sdLDL and cerebral small vessel disease (CSVD) progression in older individuals, and the influence of *Apolipoprotein E* (*APOE*) genotype on this association.

**Methods:**

Between April 2007 and July 2009, 1,143 participants ≥60 years old were recruited from the Shandong region of China, and sdLDL was measured at baseline and at each follow-up visit. White matter hyperintensities (WMHs), lacunes, microbleeds, and enlarged perivascular spaces (EPVSs) were assessed by magnetic resonance imaging. The *APOE* genotype was determined and participants were stratified as ε*4-*positive or ε*4-*negative.

**Results:**

During an average follow-up of 86.0 months, 225 participants (19.7%) developed WMH progression, 193 (16.9%) lacune progression, 170 (14.9%) microbleed progression, and 185 (16.2%) EPVS progression. Compared with patients in the first (lowest) tertile of visit-to-visit mean sdLDL, those in the second and third tertiles demonstrated significantly greater risks of WMH progression (53.5 and 105.3% higher), lacune progression (53.3 and 60.8%), microbleed progression (47.2 and 127.6%), and EPVS progression (54.0 and 135.0%) after adjustment for confounders (all adjusted *P* values for trends <0.001). Compared with patients in the first tertile of visit-to-visit sdLDL SD, those in the second and third tertiles also demonstrated significantly greater risks of WMH progression (49.9% and 143.6%), lacune progression (75.3 and 178.0%), microbleed progression (12.7 and 64.7%), and EPVS progression (41.7 and 114.6%) after adjustment (all *P* < 0.001). There were significant and positive visit-to-visit mean sdLDL × visit-to-visit sdLDL SD, visit-to-visit mean sdLD×ε*4*-positive, visit-to-visit sdLDL SD×ε*4*-positive, and visit-to-visit mean sdLDL×visit-to-visit sdLDL SD×ε*4*-positive interactions influencing CSVD progression after confounder adjustment (all *P* < 0.05).

**Conclusion:**

Large and variable visit-to-visit changes in sdLDL are independent predictors of aggressive CSVD progression, and this association is strongly influenced by *APOE* ε*4* allele genotype.

## Introduction

Cerebral small vessel disease (CSVD) is a major contributor to stroke and dementia in older individuals (1, 2). Indeed, CSVD is implicated in 25%−30% of strokes and up to 45% of dementia cases. The MRI hallmarks of CSVD are well-established, namely, white matter hyperintensities (WMHs), lacunes, microbleeds, and enlarged perivascular spaces (EPVSs) (1–3). Nonetheless, the exact etiology of CSVD is not fully understood, which has impeded the development of effective strategies for prevention and control (4).

Elevated serum low-density lipoprotein cholesterol (LDL-C) is widely regarded as the primary risk factor for macroangiopathic cardiovascular diseases, including myocardial infarction and stroke (5, 6), and several guidelines recommend LDL-C-lowering medication as the primary preventive therapy (7–9). However, the efficacy of LDL-C-lowering medications, such as statins, on CSVD progression is still controversial (10–14), possibly due to the paucity of data on the associations between various LDL-C fractions and CSVDs (15).

Low-density lipoprotein cholesterol particles are heterogeneous and fractionated based on size and density into large buoyant and small dense particles (16, 17). The small dense particles, constituting the so-called small and dense low-density lipoprotein (sdLDL) fraction, are more atherogenic than the large buoyant particles due to greater susceptibility to oxidation, higher cell membrane permeability, and reduced affinity for the LDL receptor (17, 18). Therefore, sdLDL has been suggested as a sensitive predictive biomarker for the early diagnosis of cardiovascular diseases, particularly atherosclerosis (17, 19–21). However, the association between sdLDL and CSVD progression remains unclear. In this study, our major objective was to investigate the association between CSVD progression and visit-to-visit changes in mean sdLDL among older individuals.

## Methods

### Study Participants and Design

To clarify the role of sdLDL in CSVD progression, 1,143 participants aged ≥60 years old were recruited between April 2007 and July 2009 from the Shandong area of China for a prospective and population-based cohort study (identifier at www.chictr.org.cn/, ChiCTR–EOC−17013598) (10, 22). The exclusion criteria were as follows: history of stroke/transient ischemic attack, Alzheimer's disease, Parkinson's disease, schizophrenia, seizures, claustrophobia, bipolar disorder, myocardial infarction, congestive heart failure, liver and renal diseases, dialysis treatment, drug and alcohol abuse, malignancy, contraindications to MRI, less than two annual sdLDL measurements and one brain MRI assessments during follow-up, and unwilling to provide informed consent.

The research ethics committee of the Institute of Basic Medicine, Shandong Academy of Medical Sciences, Shandong, China approved the study protocol. Each participant provided informed written consent and the study was conducted in compliance with the Declaration of Helsinki.

### Follow-UP

As previously described (10, 22), participants were examined at six-month intervals after the baseline measurements with the help of family physicians. Demographic and clinical characteristics including current medications such as antidyslipidemic, antihypertensive, antidiabetic, and antiplatelet drugs were recorded at every clinical visit. Total cholesterol (TCHO), triglyceride, high-density lipoprotein cholesterol (HDL-C), LDL-C, sdLDL, and fasting plasma glucose were assessed at baseline and at annual follow-up visits. After corresponding sdLDL assessments, respectively, white matter hyperintensities, lacunes, microbleeds, and EPVS were determined by MRI at baseline (2007–2009) and at three subsequent visits during the periods 2009–2012, 2013–2015, and 2016–2018.

### Measurements of Visit-to-Visit Mean and Variation in SdLDL

Venous blood samples were collected from each participant in the morning after overnight fasting. Blood plasma and mononuclear cells were separated and stored at −80°C for lipid assessment and *APOE* genotype determination, respectively. Plasma TCHO, triglycerides, HDL-C, LDL-C, and glucose were assessed using routine laboratory methods and sdLDL was determined using an sdLDL “Seiken” kit (Denka Seiken Co. Ltd, Tokyo, Japan) and Hitachi 7600 automatic biochemical analyzer (Hitachi, Japan) (23, 24). The participants received at least two annual sdLDL measurements during follow-up. The mean and SD in sdLDL of each participant were estimated from these serial sdLDL measurements.

### Brain MRI Assessment

Neuroimaging markers for CSVD were assessed on 3.0-Tesla scanners (GE Medical Systems, Pittsburgh, PA, USA; GE Systems, Milwaukee, WI, USA; or Siemens Medical, Erlangen, Germany) using protocols described previously (11, 22). Briefly, scans were acquired using T1-weighted 3-dimensional magnetization-prepared rapid gradient echo, T2-weighted 3-dimensional fast spin-echo, fluid-attenuated inversion recover (FLAIR), and T2^*^-weighted gradient-echo sequences. Montreal Neurological Institute templates were applied to normalize MRIs and then spatial transformation matrices were obtained. The International Consortium for Brain Mapping template for East Asian Brains was used to correct for differences in individual MRI features during the normalization. Images were then smoothed and the variability in local anatomy among subjects was minimized using a Gaussian filter.

White matter hyperintensities volume was computed from periventricular regions (frontal, parietal, occipital, and temporal), subcortical regions (frontal, parietal, occipital, and temporal), basal ganglia, and infratentorial regions on segmented T2-weighted and FLAIR axial images using FreeSurfer. The WMH-to-intracranial volume (ICV) ratio ([WMH (ml)/total intracranial volume (ml)] × 100%) was calculated to normalize individual WMH volumes. Volumetric analysis was conducted using the brain extraction tool of the FSL software package (FMRIB Software Library, Oxford, UK, www.fmrib.ox.ac.uk/fsl, version 4.19). The individual WMH pattern was graded on FLAIR images according to the Fazekas scale as none, punctuate, early confluent, and confluent.

Lacunes, microbleeds, and EPVSs were determined according to the diagnostic criteria defined in STRIVE v1 (STandards for ReportIng Vascular changes on nEuroimaging version 1). A lacune was defined as a 3–15 mm cavity with cerebrospinal-fluid-like signal intensity involving the white matter, internal capsule, basal ganglia, thalamus, or brain stem on a combination of T1-weighted, T2-weighted, and FLAIR images. Microbleeds in the brain parenchyma were defined on T2^*^-weighted images as oval or round homogenous and hypointense foci of diameter 2–10 mm. Mimics of microbleeds arising from signal averaging of bone, calcifications, and sulcal vessel signals on T2^*^-weighted images were systematically distinguished and excluded. Enlarged perivascular spaces were defined as visible fluid-filled spaces adjacent to cerebral vessels on T2-weighted and FLAIR images and distinguished from small lacunes of presumed vascular origin.

Each available scan was rated in a side-by-side fashion by experienced neuroradiologists initially blinded to clinical details, and consensus meetings were held to resolve disagreements among raters. A total of one hundred and forty randomly selected MRI scans were scored first to assess interrater and intrarater reliability. The interrater and intrarater coefficients of variation for WMH volume were 0.94 and 0.92, and the weighted Cohen's kappa values were 0.88 and 0.87 for the Fazekas scale, 0.84 and 0.83 for lacunes, 0.85 and 0.83 for microbleeds, and 0.79 and 0.80 for EPVSs, respectively, indicating good reliability.

### Identification of CSVD Progression

The progression of each CSVD neuroimaging hallmark was determined from at least two MRI assessments during follow-up. The WMH progression was assessed by volume change and visual rating. The volume change during the follow-up period was defined as the difference between each successive follow-up WMH volume measurement minus the baseline volume, while the visual rating of absence or presence of progression was assessed using the modified Rotterdam Progression scale (scores of 0 and 1, respectively) (25, 26). For lacunes, microbleeds, and EPVSs, progression was defined as the presence of any new lesions on follow-up scans (presence = 1 and absence = 0) (27). The progression of total CSVD burden was defined as the new incident of coexistence of WMH, lacune, microbleeds, and EPVSs in this study. It was rated as 1 if one of the four markers occurred, and the total score ranged from 1 to 4.

### Apolipoprotein E (APOE) Genotyping

All participants were genotyped for the *APOE* rs429358 and rs7412 single-nucleotide polymorphisms by PCR using the TaqMan genotyping kit (Applied Biosystems, Foster City, CA, USA), forward primer 5'-TTG AAG GCC TAC AAA TCG GAA CTG-3', and reverse primer 5'-CCG GCT GCC CAT CTC CAT CCG-3' (11, 28, 29). Participants with the ε*2*/ε*4*, ε*3*/ε*4*, or ε*4*/ε*4* genotype were categorized as ε*4*-positive carriers, while participants with the ε*2*/ε*2*, ε*2*/ε*3*, or ε*3*/ε*3* genotype were categorized as ε*4-*negative carriers (11, 28).

### Statistical Analysis

Participants were divided into three tertiles of visit-to-visit mean sdLDL and sdLDL SD. Variables are presented as mean (SD), median [interquartile range (IQR)], or number (percentage) as appropriate. The normality of continuous variables was determined using the Kolmogorov–Smirnov test. Mean baseline characteristics were compared among groups by one-way ANOVA with *post hoc* Bonferroni's correction, Kruskal–Wallis test with *post hoc* Wilcoxon rank-sum test, or chi-square test as indicated. Differences in the trends of WMH volume and WMH-to-ICV ratio were assessed using a linear mixed model and differences in CSVD progression risk by Kaplan–Meier analysis and log-rank test among groups. The hazard ratio (HR) with 95% CI was estimated using the Cox proportional hazards model. Models were initially adjusted for age and sex (model 1). Model 2 adjusted for smoking; alcohol consumption; the initial body mass index, blood pressure, lipids, and fasting plasma glucose at baseline; the histories of hypertension, diabetes, and dyslipidemia; medications; and the initial WMH volume (for the changes in WMH volume analysis) and WMH-to-ICV ratio (for the changes in WMH fraction analysis) at baseline base on model 1. Model 3 adjusted for visit-to-visit mean sdLDL (for the models grouped by the tertile of variability in sdLDL), variability in sdLDL (for the models grouped by the tertile of visit-to-visit mean sdLDL), and *APOE* genotype. We also conducted an exploratory investigation on the influences of visit-to-visit mean sdLDL×sdLDL SD, visit-to-visit mean sdLD×*APOE* ε*4* genotype, visit-to-visit sdLDL SD×*APOE* ε*4* genotype, and visit-to-visit mean sdLDL×sdLDL SD×*APOE* ε*4* genotype interactions on CSVD progression. Missing data were imputed using chained equations. All statistical analyses were performed using SPSS v.24.0 (SPSS Inc., Chicago, IL, USA). A *P* < 0.05 (two-tailed) was considered statistically significant for all tests.

## Results

### Baseline Characteristics

There were 1,309 initial enrolled individuals at baseline in this study. Among them, 69 failed in less than two annual sdLDL measurements and 51 failed in at least one brain MRI assessment during the follow-up period, and 46 failed to identify APOE genotype. Finally, 1,143 participants were eligible and used for further analyses. Table 1 summarizes the baseline characteristics of the eligible participants, including brain MRI parameters and *APOE* genotype. Supplementary Figure 1 presents the changes in sdLDL during the follow-up period. The median visit-to-visit mean sdLDL was 0.60 mmol/l [IQR, 0.49–0.72 mmol/l] and the median visit-to-visit sdLDL SD was 0.25 mmol/l [0.17–0.34 mmol/l]. The tertiles of visit-to-visit mean sdLDL were <0.53, 0.53–0.66, and ≥0.67 mmol/l and the tertiles for visit-to-visit sdLDL SD were <0.20, 0.20–0.30, and ≥0.31 mmol/l.

**Table 1:** Participant demographic and baseline clinical characteristics.

	**All** **(*n* = 1,143)**	**Grouped by tertile of visit-to-visit mean sdLDL**					**Grouped by tertile of visit-to-visit sdLDL SD**			
		**First tertile group** **(*n =* 384)**	**Second tertile group (*n =* 381)**	**Third tertile group (*n =* 378)**	***P*** **value**		**First tertile group (*n =* 381)**	**Second tertile group (*n =* 381)**	**Third tertile group (*n =* 381)**	***P*** **value**
**Clinical parameters**										
Age (years)	67.35 ± 5.50	67.15 ± 5.39	67.52 ± 5.59	67.46 ± 5.51	0.611		66.76 ± 5.41	67.61 ± 5.43	67.76 ± 5.60^*^	0.024
Female [*n* (%)]	652 (57.0)	196 (51.0)	205 (53.8)	251 (66.4) ^*^^†^	<0.001		217 (57.0)	214 (56.2)	221 (58.0)	0.876
Smoking [*n* (%)]	368 (32.2)	96 (25.0)	125 (32.8)^*^	147 (38.9)^*^	<0.001		109 (28.6)	122 (32.0)	137 (36.0)	0.094
Alcohol consumption [*n* (%)]	382 (33.4)	109 (28.4)	125 (32.8)	148 (39.2)^*^	0.007		107 (28.1)	138 (36.2)^*^	137 (36.0)^*^	0.026
Hypertension [*n* (%)]	797 (69.7)	256 (66.7)	263 (69.0)	278 (73.5)	0.111		266 (69.8)	257 (67.5)	274 (71.9)	0.407
Antihypertensive medication [*n* (%)]	624 (54.6)	214 (55.7)	209 (54.9)	201 (53.2)	0.772		212 (55.6)	208 (54.6)	204 (53.5)	0.844
Diabetes [*n* (%)]	172 (15.0)	48 (12.5)	61 (16.0)	63 (16.7)	0.223		53 (13.9)	55 (14.4)	64 (16.8)	0.494
Anti-diabetes medication [*n* (%)]	159 (13.9)	56 (14.6)	54 (14.2)	49 (13.0)	0.789		59 (15.5)	51 (13.4)	49 (12.9)	0.541
Dyslipidemia [*n* (%)]	329 (28.8)	103 (26.8)	107 (28.1)	119 (31.5)	0.341		104 (27.3)	104 (27.3)	121 (31.8)	0.291
Antidyslipidemia medication [*n* (%)]	80 (7.0)	31 (8.1)	26 (6.8)	23 (6.1)	0.553		31 (8.1)	28 (7.3)	21 (5.5)	0.346
Antiplatelet medication [*n* (%)]	99 (8.7)	38 (9.9)	32 (8.4)	29 (7.7)	0.538		34 (8.9)	33 (8.7)	32 (8.4)	0.967
Heart rate (bpm)	70.12 ± 8.11	69.70 ± 8.12	69.86 ± 7.49	70.81 ± 8.67	0.122		69.13 ± 8.25	70.38 ± 8.16	70.86 ± 7.86^*^	0.009
Body mass index (kg/m^2^)	24.94 ± 2.39	24.86 ± 2.46	24.71 ± 2.43	24.94 ± 2.39^†^	0.006		25.00 ± 2.35	24.78 ± 2.45	25.03 ± 2.37	0.304
**Blood pressure (mm Hg)**										
Systolic blood pressure	146.32 ± 15.53	146.13 ± 15.96	145.51 ± 15.09	147.34 ± 15.52	0.257		144.09 ± 15.72	146.52 ± 15.22	148.35 ± 15.39^*^	0.001
Diastolic blood pressure	76.69 ± 8.08	76.80 ± 7.69	76.38 ± 8.35	76.89 ± 8.21	0.651		76.04 ± 8.15	76.77 ± 7.78	77.26 ± 8.28	0.112
**Biochemical parameters (mmol/L)**										
Total cholesterol	4.72 ± 0.75	4.37 ± 0.70	4.69 ± 0.69^*^	5.09 ± 0.69^*^,^†^	<0.001		4.52 ± 0.75	4.77 ± 0.73^*^	4.86 ± 0.74^*^	<0.001
Triglycerides	1.60 ± 0.52	1.54 ± 0.52	1.57 ± 0.50	1.68 ± 0.54^*^,^†^	0.001		1.57 ± 0.51	1.61 ± 0.52	1.61 ± 0.54	0.361
HDL-C	1.16 ± 0.42	1.27 ± 0.49	1.11 ± 0.38^*^	1.09 ± 0.37^*^	<0.001		1.20 ± 0.45	1.16 ± 0.42	1.11 ± 0.40^*^	0.019
LDL-C	2.83 ± 0.69	2.41 ± 0.62	2.86 ± 0.60^*^	3.24 ± 0.57^*^^†^	<0.001		2.61 ± 0.68	2.88 ± 0.64^*^	3.02 ± 0.69^*^^†^	<0.001
sdLDL	0.64 (0.44, 0.89)	0.47 (0.33, 0.65)	0.63 (0.46, 0.81)^*^	0.89 (0.67, 1.09)^*^,^†^	<0.001		0.56 (0.42, 0.72)	0.69 (0.44, 0.90)^*^	0.75 (0.46, 1.04)^*^^†^	<0.001
FPG	5.66 ± 1.49	5.61 ± 1.41	5.63 ± 1.42	5.76 ± 1.64	0.321		5.63 ± 1.42	5.64 ± 1.47	5.73 ± 1.58	0.545
**Brain magnetic resonance imaging**										
WMH volume (mL)	4.69 (3.16, 6.16)	4.19 (2.68, 5.59)	4.63 (3.18, 6.18)^*^	5.22 (3.81, 6.67)^*^^†^	<0.001		4.09 (2.72, 5.47)	4.56 (3.06, 5.93)^*^	5.55 (3.92, 6.95)^*^^†^	<0.001
WMH-to-ICV ratio (%)	0.36 (0.24, 0.48)	0.31 (0.21, 0.45)	0.36 (0.25, 0.48)^*^	0.40 (0.30, 0.52)^*^^†^	<0.001		0.31 (0.21, 0.43)	0.35 (0.24, 0.45)^*^	0.44 (0.31, 0.54)^*^^†^	<0.001
Incidence of Fazekas scale ≥2 [*n* (%)]	114 (10.0)	27 (7.0)	33 (8.7)	54 (14.3)^*^^†^	0.002		19 (5.0)	29 (7.6)	66 (17.3)^*^^†^	<0.001
Incidence of lacunes [*n* (%)]	96 (8.4)	24 (6.3)	28 (7.3)	44 (11.6)^*^^†^	0.018		15 (3.9)	23 (6.0)	58 (15.2)^*^^†^	<0.001
Incidence of microbleeds [*n* (%)]	71 (6.2)	16 (4.2)	19 (5.0)	36 (9.5)^*^^†^	0.004		16 (4.2)	19 (5.0)	36 (9.4)^*^^†^	0.005
Incidence of EPVS [*n* (%)]	124 (10.8)	38 (9.9)	36 (9.4)	50 (13.2)	0.188		36 (9.4)	40 (10.5)	48 (12.6)	0.363
***APOE*** **genotype**										
*ε4*-positive carriers [*n* (%)]	297 (26.0)	80 (20.8)	105 (27.6)^*^	112 (29.6)^*^	0.015		81 (21.3)	100 (26.2)	116 (30.4)^*^	0.015

*Compared with the First tertile group*.

* *P < 0.05; compared with the Second tertile group*,

† *P < 0.05*.

*sdLDL, small and dense low-density lipoproteins; SD, standard deviation; HDL-c, high-density lipoprotein cholesterol; LDL-c, low-density lipoprotein cholesterol; FPG, fasting plasma glucose; WMH, white matter hyperintensities; ICV, intracranial volume; EPVS, enlarged perivascular space; APOE, apolipoprotein E*.

### Outcomes

During an average 86.0 [IQR, 84.0–90.0] months of follow-up, WMH volume increased by 1.72 [IQR, 1.44–2.03] ml and WMH-to-ICV ratio by 0.14% [IQR, 0.11%−0.17%]. During follow-up, 225 participants (19.7%) developed WMH progression, 193 (16.9%) lacune progression, 170 (14.9%) microbleed progression, and 185 (16.2%) developed EPVS progression. The average of CSVD new burden was 0.67 (IQR, 0–1).

### Contributions of Visit-to-Visit Mean SdLDL to CSVD Progression Risk

**Figure 1:**
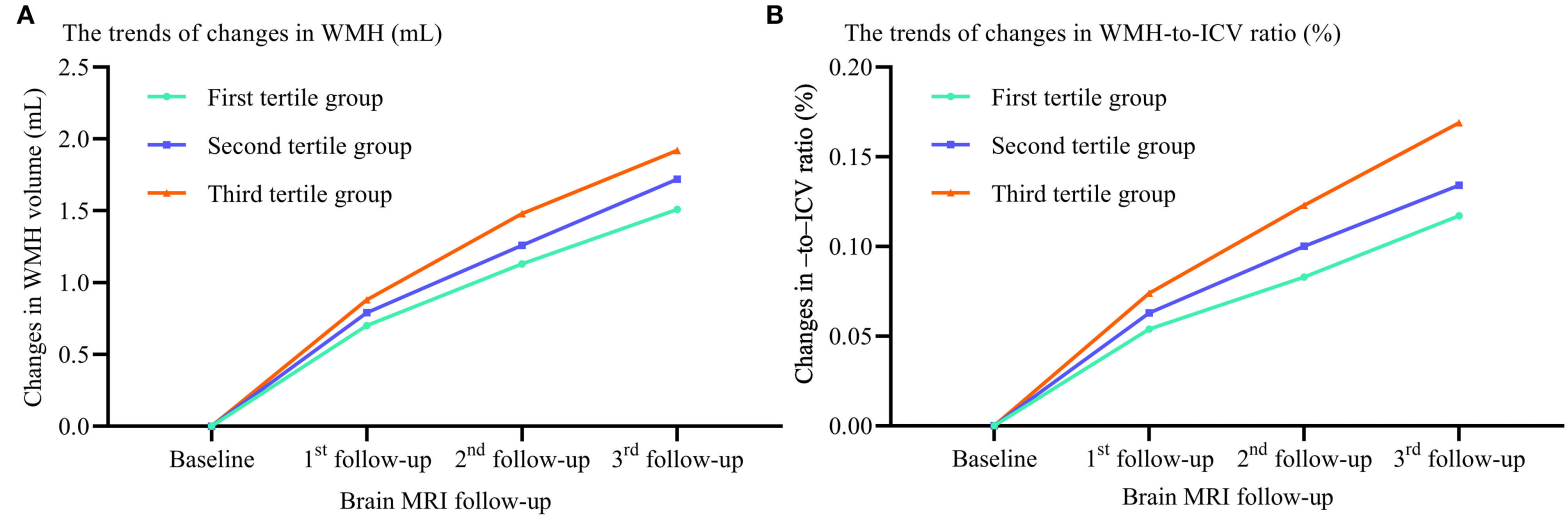
Differences in WMH volume and WMH-to-ICV ratio changes among tertile groups stratified by visit-to-visit mean sdLDL during the follow-up period. **(A)** Changes in WMH. **(B)** Changes in WMH-to-ICV ratio. WMH, white matter hyperintensities; ICV, intracranial volume.

**Figure 2:**
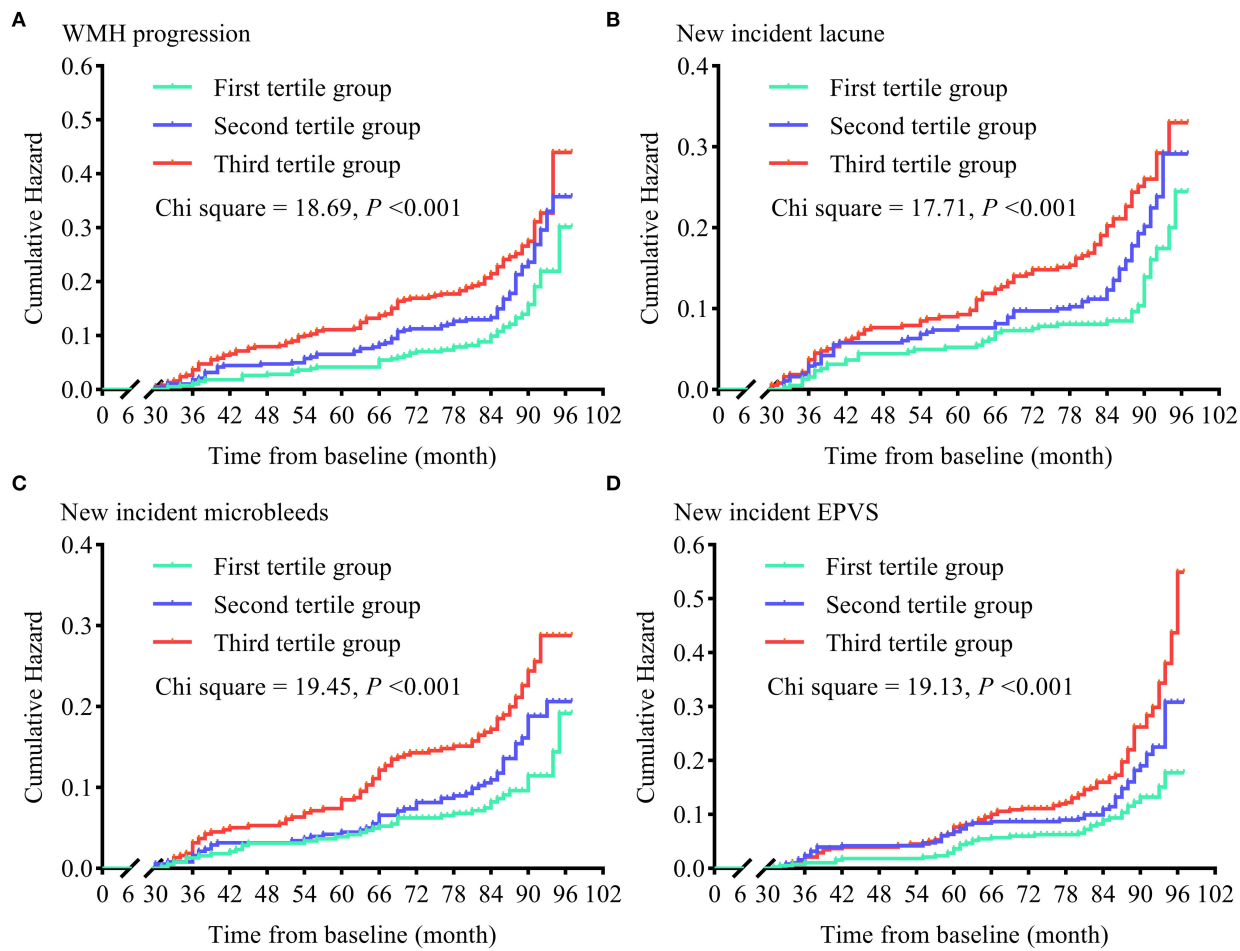
Cumulative hazards of cerebral small vessel disease progression in tertile groups stratified by visit-to-visit mean sdLDL during the follow-up period. **(A)** Cumulative hazards of WMH progression. **(B)** Cumulative hazards of new-incident lacunes. **(C)** Cumulative hazards of new-incident microbleeds. **(D)** Cumulative hazards of new-incident EPVS. WMH, white matter hyperintensities; EPVS, enlarged perivascular space.

To examine the contributions of sdLDL to CSVD progression rise, we first compared the trends in WMH volume and WMH-to-ICV ratio changes and CSVD new burden among visit-to-visit mean sdLDL tertile groups (defined in the previous section) and found that compared to the first tertile group, the second and third groups demonstrated significantly greater increases in WMH volume, WMH-to-ICV ratio, and CSVD new burden (all *P* < 0.05). Furthermore, increases in WMH volume, WMH-to-ICV ratio, and CSVD new burden were significantly greater in the third tertile group than in the second (all *P* < 0.05), and these differences remained significant after values were adjusted for confounders including baseline WMH volume and baseline WMH-to-ICV ratio (adjusted *P* values for trends <0.05, Figure 1 and Supplementary Figure 2). Visit-to-visit increases in mean sdLDL were also associated with greater risks of WMH, lacune, microbleed, and EPVS progression. Compared with the first tertile group, the second and third tertile groups showed significantly greater risks of WMH progression (53.5% and 105.3% greater), lacune progression (53.3% and 60.8%), microbleed progression (47.2% and 127.6%), and EPVS progression (54.0% and 135.0%) (all adjusted *P* values for trends <0.001). The HRs and 95% CIs resulting from analysis model 3 are provided in Figure 2 and Supplementary Table 1.

### Contributions of Visit-to-Visit SdLDL Variability to CSVD Progression Risk

**Figure 3:**
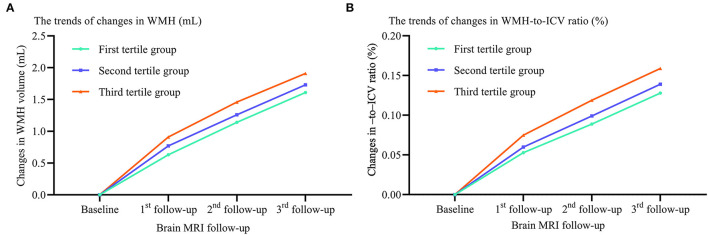
Differences in WMH volume and WMH-to-ICV ratio changes among tertile groups stratified by visit-to-visit sdLDL SD during the follow-up period. **(A)** Changes in WMH. **(B)** Changes in WMH-to-ICV ratio. WMH, white matter hyperintensities; ICV, intracranial volume.

We also compared the trends in WMH volume and WMH-to-ICV ratio changes and CSVD new burden among tertile groups stratified by the visit-to-visit sdLDL SD, and again found the WMH volume and WMH-to-ICV ratio changes and CSVD new burden were significantly greater in the third tertile group than in the first and second tertile groups, and greater in the second than the first tertile group (all *P* < 0.05). Furthermore, these differences among tertile groups remained significant after adjustment for confounders including the baseline WMH volume and WMH-to-ICV ratio (all adjusted *P* values for trend <0.05, Figure 3).

**Figure 4:**
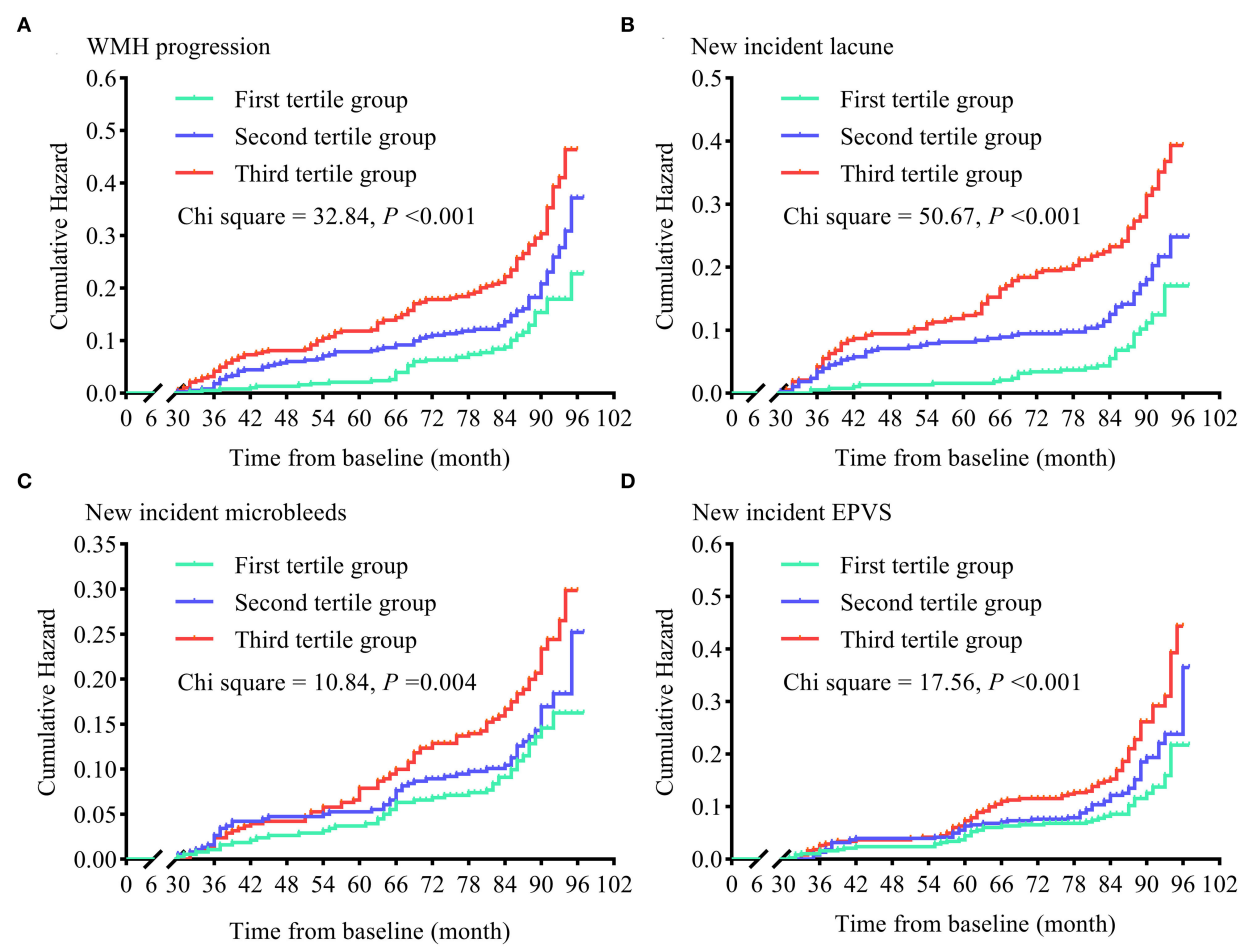
Cumulative hazards of cerebral small vessel disease progression in tertile groups stratified by visit-to-visit sdLDL SD during the follow-up period. **(A)** Cumulative hazards of WMH progression. **(B)** Cumulative hazards of new-incident lacunes. **(C)** Cumulative hazards of new-incident microbleeds. **(D)** Cumulative hazards of new-incident EPVS. WMH, white matter hyperintensities; EPVS, enlarged perivascular space.

Like increased mean sdLDL, greater visit-to-visit sdLDL SD was associated with significantly higher risks of WMH progression (49.9% higher in the second tertile and 143.6% higher in the third tertile group compared with the first), lacune progression (75.3 and 178.0% higher, respectively), microbleed progression (12.7 and 64.7% higher, respectively), and EPVS progression (41.7 and 114.6% higher, respectively), and these increases were still significant after adjustment for confounders (all adjusted *P* values for trends <0.001). The HRs and 95% CIs resulting from analysis model 3 are provided in Figure 4 and Supplementary Table 1.

### Contributions of Interactions Among Visit-to-Visit Mean SdLDL, Visit-to-Visit SdLDL Variability, and *APOE* Genotype to CSVD Progression Risk

We also identified significant positive visit-to-visit mean sdLDL×visit-to-visit sdLDL SD, visit-to-visit mean sdLDL×*APOE* ε*4* allele, visit-to-visit sdLDL SD×*APOE* ε*4* allele, and visit-to-visit mean sdLDL×visit-to-visit sdLDL SD×*APOE* ε*4* allele interaction effects on CSVD progression after adjustment for confounders (adjusted *P* values <0.05). The details are shown in Table 2.

**Table 2:** Interactions among visit-to-visit mean sdLDL, visit-to-visit sdLDL variability, and *APOE* genotype influencing cerebral small vessel disease progression.

	**WMH progression**		**Lacune progression**		**Microbleed progression**		**EPVS progression**	
	**HR (95% CI)**	***P*** **value**	**HR (95% CI)**	***P*** **value**	**HR (95% CI)**	***P*** **value**	**HR (95% CI)**	***P*** **value**
**Model 1**								
Visit-to-visit mean sdLDL× visit-to-visit sdLDL SD	1.173 (1.113,1.236)	<0.001	1.197 (1.131, 1.266)	<0.001	1.141 (1.074, 1.212)	<0.001	1.191 (1.120, 1.267)	<0.001
Visit-to-visit mean sdLDL ×*APOE ε4* genotype	1.509 (1.359, 1.675)	<0.001	1.456 (1.300, 1.631)	<0.001	1.593 (1.416, 1.792)	<0.001	1.349 (1.191, 1.527)	<0.001
Visit-to-visit sdLDL SD ×*APOE ε4* genotype	1.497 (1.351, 1.695)	<0.001	1.434 (1.282, 1.605)	<0.001	1.576 (1.395, 1.781)	<0.001	1.298 (1.147, 1.469)	<0.001
Visit-to-visit mean sdLDL× visit-to-visit sdLDL SD ×*APOE ε4* genotype	1.159 (1.115, 1.206)	<0.001	1.147 (1.098, 1.197)	<0.001	1.186 (1.136, 1.239)	<0.001	1.121 (1.066, 1.178)	<0.001
**Model 2**								
Visit-to-visit mean sdLDL×visit-to-visit sdLDL SD	1.145 (1.094, 1.198)	<0.001	1.176 (1.120, 1.235)	<0.001	1.132 (1.074, 1.193)	<0.001	1.178 (1.111, 1.248)	<0.001
Visit-to-visit mean sdLDL×*APOE ε4* genotype	1.504 (1.350, 1.674)	<0.001	1.417 (1.262, 1.591)	<0.001	1.569 (1.396, 1.763)	<0.001	1.349 (1.190, 1.530)	<0.001
Visit-to-visit sdLDL SD × *APOE ε4* genotype	1.500 (1.348, 1.669)	<0.001	1.421 (1.265, 1.595)	<0.001	1.575 (1.394, 1.778)	<0.001	1.298 (1.145, 1.472)	<0.001
Visit-to-visit mean sdLDL×visit-to-visit sdLDL SD ×*APOEε4*genotype	1.160 (1.113, 1.210)	<0.001	1.140 (1.090, 1.193)	<0.001	1.188 (1.134, 1.245)	<0.001	1.120 (1.067, 1.176)	<0.001
**Model 3**								
Visit-to-visit mean sdLDL×visit-to-visit sdLDL SD	1.088 (1.030, 1.149)	<0.001	1.063 (1.003, 1.127)	0.039	1.103 (1.035, 1.175)	0.003	1.139 (1.083, 1.197)	<0.001
Visit-to-visit mean sdLDL ×*APOE ε4* genotype	1.431 (1.283, 1.596)	<0.001	1.294 (1.150, 1.456)	<0.001	1.510 (1.334, 1.708)	<0.001	1.341 (1.188, 1.513)	<0.001
Visit-to-visit sdLDL SD ×*APOE ε4* genotype	1.396 (1.255, 1.554)	<0.001	1.259 (1.121, 1.413)	<0.001	1.488 (1.315, 1.683)	<0.001	1.286 (1.140, 1.451)	<0.001
Visit-to-visit mean sdLDL×visit-to-visit sdLDL SD ×*APOE ε4* genotype	1.127 (1.081, 1.175)	<0.001	1.083 (1.035, 1.133)	0.001	1.167 (1.113, 1.224)	<0.001	1.114 (1.063, 1.167)	<0.001

*Model 1 is adjusted for age and sex. Model 2 is adjusted for smoking, alcohol consumption, initial body mass index, blood pressure, serum lipids, and fasting plasma glucose at baseline, and either the initial WMH volume (for WMH volume change analysis) and WMH-to-ICV ratio (for WMH-to-ICV ratio change analysis) at baseline. Model 3 is adjusted for visit-to-visit mean sdLDL (for the models grouped by tertile of sdLDL variability), variability in sdLDL (for the models grouped by visit-to-visit mean sdLDL tertile), and APOE genotype. WMH, white matter hyperintensities; EPVS, enlarged perivascular space; HR, hazard ratio; sdLDL, small and dense low-density lipoproteins; SD, standard deviation*.

## Discussion

In this prospective longitudinal cohort study, we found that visit-to-visit mean sdLDL and visit-to-visit sdLDL SD were independently associated with the risk of CSVD progression over a mean duration of 86.0 months in older adults. Specifically, greater visit-to-visit mean sdLDL and sdLDL SD predicted more aggressive progression of the CSVD manifestations WMH volume, lacunes, microbleeds, and EPVS. Furthermore, there were significant positive mutual interaction effects of visit-to-visit mean sdLDL and visit-to-visit sdLDL SD and positive interaction effects of both with *APOE* ε*4* genotype on CSVD progression.

The sdLDL faction of LDL is strongly associated with atherosclerotic disease (19, 20, 30, 31), possibly due to the greater susceptibility of sdLDL particles to oxidation, higher cell membrane permeability than other fractions, and lower LDL receptor affinity (17, 18). Several reports have document associations between sdLDL level and both carotid artery intima-media thickness and plaque progression (32–34). The Atherosclerosis Risk in Communities study also found that the risk of coronary artery disease was 1.5-fold higher in individuals with sdLDL in the four quartiles (≥75th percentile) compared to the lowest quartile (20), while a Chinese cohort study with an average 9.5-year follow-up identified sdLDL level as an independent risk factor for major adverse cardiovascular events in hypertensive subjects (24). Here, we extend these findings by demonstrating a significant association of sdLDL with CSVD. To the best of our knowledge, only one previous study has reported an association between sdLDL and a CSVD sign (brain WMH volume) (35) but the cross-sectional design precluded evaluation of an association with disease progression. In the current prospective longitudinal cohort study, we show that long-term sdLDL elevation and greater variability are strongly associated with CSVD progression, particularly in *APOE* ε*4* carriers. We also found that greater visit-to-visit mean sdLDL and sdLDL SD were associated with higher risks of lacune, microbleed, and EPVS progression and WMH volume progression, even after adjustment for multiple confounders including baseline sdLDL, LDL-C, and WMH volume and also lacune, microbleed, and EPVS incidence.

Serum lipid levels and sdLDL levels are influenced by numerous factors, namely, diet, exercise level, medication adherence and dose, season, and mood (18, 36–40). Relationships between high serum LDL-C variability and increased cardiovascular and cerebrovascular disease risks are well established (36, 41–43). Thus, we hypothesized that sdLDL variability would also be closely associated with CSVD progressions, and indeed higher tertile of sdLDL SD predicted WMH, lacune, microbleed, and EPVS progression after adjustment for confounders including visit-to-visit mean sdLDL. In addition, visit-to-visit sdLDL SD interacted synergistically with visit-to-visit mean sdLDL to further increase CSVD risk.

One of the major functions of *APOE* is to regulate lipid metabolism, especially of TCHO and LDL-C (11, 28, 44). Apolipoprotein E is abundantly expressed in the brain and accumulation on vessel walls is strongly associated with CSVD severity (45–47). Our exploratory analysis showed that the *APOE* ε*4* allele significantly and positively interacted with both higher visit-to-visit mean sdLDL and visit-to-visit sdLDL SD to enhance CSVD progressions risk. Thus, the *APOE* genotype is an important mediator of the association between serum sdLDL and CSVD progression in older individuals.

The major strengths of this study include the prospective longitudinal cohort design with long-term follow-up and a large sample size. In addition, we examined the effects of both higher long-term mean sdLDL and greater long-term sdLDL variability on CSVD progression and the interaction between these factors and *APOE* genotype. On the other hand, many critical confounders were not examined, such as lifestyle, diet, season, mood changes, and medication adherence, all of which can significantly influence serum lipid levels and variability (18, 37–40). Second, all participants were of Han ethnicity, so applicability to other ethnicities is uncertain. Third, we did not examine many additional pathogenic factors that could directly influence disease progression or the effects of serum sdLDL, such as oxidative stress, endothelial dysfunction, and inflammatory status.

In conclusion, serum sdLDL level and variation are critical independent and synergistically acting risk factors for CSVD progression in older individuals. Moreover, the *APOE* genotype strongly influences the association of sdLDL level with CSVD progression. However, further multinational studies involving additional ethnic groups and controlling for factors such as lifestyle, diet, and medication adherence are needed to validate our results.

## Data Availability Statement

The original contributions presented in the study are included in the article/Supplementary Material, further inquiries can be directed to the corresponding authors.

## Ethics Statement

The studies involving human participants were reviewed and approved by the Research Ethics Committee of the Institute of Basic Medicine, Shandong Academy of Medical Sciences, Shandong, China. The patients/participants provided their written informed consent to participate in this study.

## Author Contributions

WL wrote the manuscript with input from all authors. WL, JX, HS, CZ, YY, and HZ were responsible for data collection. WL and HZ performed the statistical analysis. Y-CL and ZL designed and supervised the study, drafting, and editing of the manuscript. All authors contributed to a critical review of the manuscript.

## Funding

This work was supported by the National Natural Science Foundation of China (Grant Nos. 81870281, 81670432, and 81973139), the Zhejiang Provincial Natural Science Foundation of China (Grant Nos. LY18H020011 and LQ19H020005), the Key Technology Research and Development Project of Shandong Province (Grant Nos. 2019GSF108079 and 2018GSF118044), the Medical and Health Science and Technology Development Plan Project of Shandong, China (Grant No. 202103010686), and the Innovation Project of Shandong Academy of Medical Sciences, and the Academic Promotion Program of Shandong First Medical University.

## Conflict of Interest

The authors declare that the research was conducted in the absence of any commercial or financial relationships that could be construed as a potential conflict of interest. The reviewer CY declared a shared parent affiliation with the authors HS, CZ, HZ, YY, and ZL to the handling editor at the time of review.

## Publisher's Note

All claims expressed in this article are solely those of the authors and do not necessarily represent those of their affiliated organizations, or those of the publisher, the editors and the reviewers. Any product that may be evaluated in this article, or claim that may be made by its manufacturer, is not guaranteed or endorsed by the publisher.

## Abbreviations

APOE: Apolipoprotein E
CSVD: cerebral small vessel disease
EPVS: enlarged perivascular spaces
FLAIR: fluid-attenuated inversion recovery
HDL-C: high-density lipoprotein cholesterol
ICV: intracranial volume
LDL-C: low-density lipoprotein cholesterol
sdLDL: small and dense low-density lipoprotein
TCHO: total cholesterol
WMH: white matter hyperintensities.
