# Supplementary material for: Excessive Visit-to-Visit Small and Dense Low-Density Lipoproteins Elevate Cerebral Small Vessel Disease Progression Risk in the Elderly
Source: Front Neurol. 2022 Jun 29;13:851735. doi: 10.3389/fneur.2022.851735 (PMC9277007; doi:10.3389/fneur.2022.851735)
Supplement: Supplementary file 1 [file Data_Sheet_1.pdf]

Supplementary Material:

Title: Excessive visit-to-visit small and dense low-density lipoproteins elevate cerebral small vessel disease progression risk in the elderly

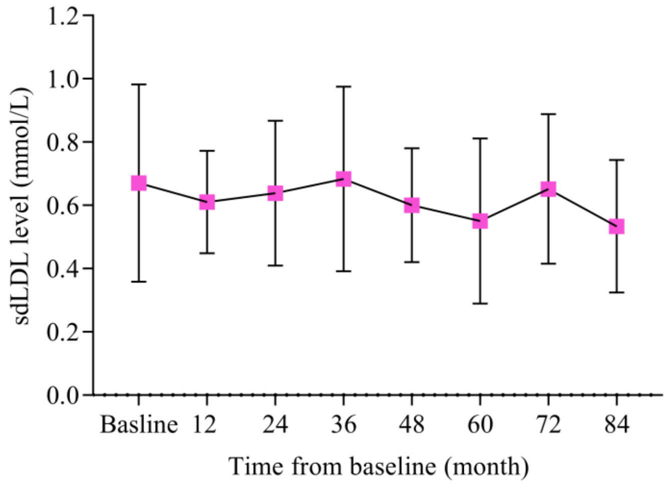

Supplementary Figure 1. The changes in sdLDL during the follow-up period

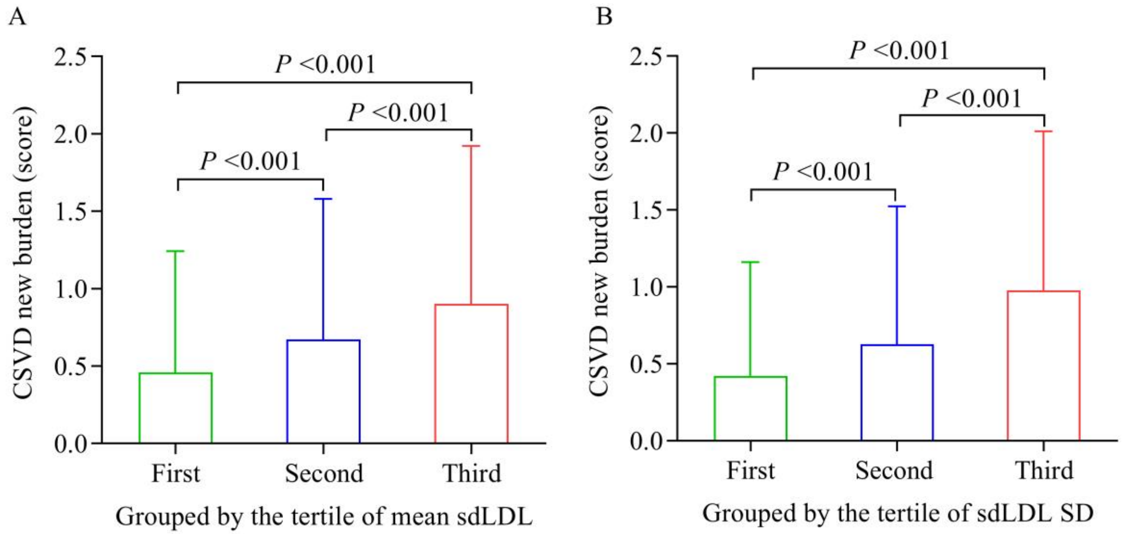

Supplementary Figure 2. The differences in CSVD new burden during the follow-up period among groups. A grouped by the tertile of visit-to-visit mean sdLDL. B grouped by the tertile of visit-to-visit sdLDL SD.

Supplementary Table 1. Contributions of small and dense low density lipoproteins to cerebral small vessel disease progression

	WMH progression			Lacune progression			Microbleed progression			EPVS progression		
	Events (*n*)	HR (95% CI)	*P* value	Events (*n*)	HR (95% CI)	*P* value	Events (*n*)	HR (95% CI)	*P* value	Events (*n*)	HR (95% CI)	*P* value
**Grouped by the tertile of visit-to-visit mean of sdLDL**												
Model 1												
First tertile group(*n*=384)	53	1 [Ref.]		44	1 [Ref.]		38	1 [Ref.]		41	1 [Ref.]	
Second tertile group (*n*=381)	77	1.836 (1.268, 2.658)	0.001	65	1.689 (1.130, 2.524)	0.011	54	1.631 (1.052, 2.531)	0.029	60	1.690 (1.110, 2.574)	0.014
Third tertile group (*n*=378)	95	2.723 (1.830, 4.051)	<0.001	84	2.101 (1.454, 3.036)	<0.001	78	2.714 (1.724, 4.271)	<0.001	84	2.781 (1.786, 4.329)	<0.001
P value for trend		<0.001			<0.001			<0.001			<0.001	
Model 2												
First tertile group (*n*=384)	53	1 [Ref.]		44	1 [Ref.]		38	1 [Ref.]		41	1 [Ref.]	
Second tertile	77	1.677 (1.158,	0.006	65	1.543 (1.052,	0.026	54	1.577 (1.016,	0.042	60	1.627 (1.068,	0.023

group (*n*=381)		2.430)			2.263)			2.449)			2.479)	
Third tertile group (*n*=378)	95	2.267 (1.512, 3.398)	<0.001	84	1.926 (1.266, 2.928)	0.002	78	2.532 (1.597, 4.014)	<0.001	84	2.557 (1.630, 4.011)	<0.001
P value for trend		<0.001			0.007			<0.001			<0.001	
Model 3												
Firsttertile group (*n*=384)	53	1 [Ref.]		44	1 [Ref.]		38	1 [Ref.]		41	1 [Ref.]	
Second tertile group (*n*=381)	77	1.535 (1.082, 2.179)	0.016	65	1.533 (1.040, 2.260)	0.031	54	1.472 (0.972, 2.230)	0.068	60	1.540 (1.035, 2.292)	0.033
Third tertile group (*n*=378)	95	2.053 (1.462, 2.883)	<0.001	84	1.608 (1.052, 2.458)	0.028	78	2.276 (1.538, 3.368)	<0.001	84	2.350 (1.612, 3.427)	<0.001
P value for trend		<0.001			0.036			<0.001			<0.001	
**Grouped by the tertile of visit-to-visit SD in sdLDL**												
Model 1												
First tertile group (*n*=381)	46	1 [Ref.]		31	1 [Ref.]		43	1 [Ref.]		41	1 [Ref.]	
Second tertile	70	1.549 (1.067,	0.021	58	1.894 (1.224,	0.004	52	1.201 (0.801,	0.375	59	1.444 (0.968,	0.071

group (*n*=381)		2.248)			2.931)			1.800)			2.153)	
Third tertile group (*n*=381)	109	2.582 (1.825, 3.652)	<0.001	104	3.612 (2.415, 5.403)	<0.001	75	1.762 (1.207, 2.571)	0.003	85	2.204 (1.514, 3.209)	<0.001
P value for trend		<0.001			<0.001			0.008			<0.001	
Model 2												
First tertile group (*n*=381)	46	1 [Ref.]		31	1 [Ref.]		43	1 [Ref.]		41	1 [Ref.]	
Second tertile group (*n*=381)	70	1.585 (1.085, 2.315)	0.017	58	1.815 (1.165, 2.828)	0.008	52	1.182 (0.782, 1.788)	0.428	59	1.439 (0.958, 2.161)	0.079
Third tertile group (*n*=381)	109	2.773 (1.928, 3.988)	<0.001	104	2.945 (1.926, 4.503)	<0.001	75	1.764 (1.186, 2.622)	0.005	85	2.199 (1.482, 3.263)	<0.001
P value for trend		<0.001			<0.001			0.010			<0.001	
Model 3												
First tertile group (*n*=381)	46	1 [Ref.]		31	1 [Ref.]		43	1 [Ref.]		41	1 [Ref.]	
Second tertile group (*n*=381)	70	1.499 (1.024, 2.193)	0.037	58	1.753 (1.122, 2.738)	0.014	52	1.127 (0.743, 1.709)	0.574	59	1.417 (0.941, 2.134)	0.095

Third tertile group(n=381)	109	2.436 (1.686, 3.520)	<0.001	104	2.780 (1.807, 4.277)	<0.001	75	1.647 (1.102, 2.460)	0.015	85	2.146 (1.438, 3.203)	<0.001
P value for trend		<0.001			<0.001			0.026			0.001	

Model 1 adjusted for age, sex. Model 2 adjusted for smoking, alcohol consumption, and the initial body mass index, blood pressure, lipids, and fasting plasma glucose at baseline, and the initial WMH volume (for the changes in WMH volume analysis) and WMH-to-ICV ratio (for the changes in WMH fraction analysis) at baseline base on Model 1. Model 3 adjusted for visit-to-visit mean of sdLDL (for the Models grouped by the tertile of variability in sdLDL), variability in sdLDL (for the Models grouped by the tertile of visit-to-visit mean of sdLDL), and *APOE* genotype.WMH indicates white matter hyperintensities; EPVS, enlarged perivascular space; HR, hazard ratio; sdLDL, small and dense low density lipoproteins; SD, standard deviation.
